# Diapause and overwintering of two spruce bark beetle species

**DOI:** 10.1111/phen.12200

**Published:** 2017-07-07

**Authors:** Martin Schebeck, E. Matthew Hansen, Axel Schopf, Gregory J. Ragland, Christian Stauffer, Barbara J. Bentz

**Affiliations:** ^1^ Department of Forest and Soil Sciences Boku, University of Natural Resources and Life Sciences Vienna Austria; ^2^ Rocky Mountain Research Station USDA Forest Service Logan Utah U.S.A.; ^3^ Department of Integrative Biology University of Colorado Denver Denver Colorado U.S.A.

**Keywords:** Dendroctonus rufipennis, diapause, Ips typographus, overwintering, Scolytines, voltinism

## Abstract

Diapause, a strategy to endure unfavourable conditions (e.g. cold winters) is commonly found in ectothermic organisms and is characterized by an arrest of development and reproduction, a reduction of metabolic rate, and an increased resistance to adversity. Diapause, in addition to adaptations for surviving low winter temperatures, significantly influences phenology, voltinism and ultimately population growth. We review the literature on diapause and overwintering behaviour of two bark beetle species that affect spruce‐dominated forests in the northern hemisphere, and describe and compare how these strategies can influence population dynamics. The European spruce bark beetle Ips typographus (L.) (Coleoptera, Curculionidae) is the most important forest pest of Norway spruce in Europe. It enters an adult reproductive diapause that might be either facultative or obligate. Obligate diapausing beetles are considered strictly univoltine, entering this dormancy type regardless of environmental cues. Facultative diapausing individuals enter diapause induced by photoperiod, modified by temperature, thus being potentially multivoltine. The spruce beetle Dendroctonus rufipennis (Kirby) (Coleoptera: Curculionidae) infests all spruce species in its natural range in North America. A facultative prepupal diapause is averted by relatively warm temperatures, resulting in a univoltine life cycle, whereas cool temperatures induce prepupal diapause leading to a semivoltine cycle. An adult obligate diapause in D. rufipennis could limit bi‐ or multivoltinism. We discuss and compare the influence of diapause and overwinter survival on voltinism and population dynamics of these two species in a changing climate and provide an outlook on future research.

## Introduction

Diapause is a widespread strategy used by insects to overcome harsh conditions. An arrest of reproduction and direct development, a suppression of metabolism and an increased stress resistance are main characteristics of this dormancy type (Lees, 1956; Tauber & Tauber, 1976; Tauber *et al*., 1986; Danks, 1987; Denlinger, 2002; Koštál, 2006). Diapause can also affect phenology, and the response to diapause‐inducing cues that can vary geographically results in variability in voltinism (i.e. the number of generations per year) among and within species (Bentz & Jönsson, 2015). The number of generations produced per year greatly influences the growth and dynamics of insect populations, which are two key factors in the management of economically important forest insect species.

Bark beetles (Coleoptera: Curculionidae, Scolytinae) are among the most important forest pests globally. This weevil subfamily comprises approximately 6000 species of bark and wood boring insects worldwide, a group with a wide diversity of life histories. This includes, for example, adaptations related to reproductive behaviour, feeding modes and interactions with other organisms. Bark beetles are associated with a variety of plant taxa and their life cycles are closely tied to their hosts. Scolytines spend major parts of their life inside plants that serve as a food source, habitat for mating, development and overwintering (Wood, 1982; Kirkendall, 1983; Pfeffer, 1995; Knižek & Beaver, 2004; Bentz & Jönsson, 2015; Hulcr *et al*., 2015). Bark beetles play important roles in forest ecosystems. They affect nutrient cycles, alter stand structure and species composition, and drive successional processes (Hansen, 2014). Moreover, their associations with other organisms, such as fungi or bacteria, contribute to biodiversity (Hofstetter *et al*., 2015). Several species, including those within *Dendroctonus* and *Ips* genera, can cause extensive tree mortality with significant ecological and economic impacts (Weed *et al*., 2013; Cognato, 2015; Raffa *et al*., 2015; Six & Bracewell, 2015).

Bark beetles have a particularly large influence on trees in the genus *Picea*, an important component of conifer forests in the northern hemisphere. The European spruce bark beetle *Ips typographus* (L.) (Coleoptera: Curculionidae) has a palearctic distribution (Cognato, 2015) and is the most important forest pest of Norway spruce, *Picea abies*, in Europe (Wermelinger, 2004). The spruce beetle *Dendroctonus rufipennis* (Kirby) (Coleoptera: Curculionidae) infests all spruce found within its range in North America, most notably *Picea glauca*, *Picea* x *lutzii*, *Picea sitchensis* and *Picea engelmannii* (Holsten *et al*., 1999). Although in different genera, these two species of spruce bark beetle can have similar impacts on their host tree ecosystems.

The life histories of *I. typographus* and *D. rufipennis* are strongly influenced by seasonal events and they respond with similar physiological strategies. For example, both species enter diapause to buffer against harsh winter conditions and to synchronize life cycles, a widespread strategy in insects that is described for multiple bark beetle species (Ryan, 1959; Scott & Berryman, 1972; Birch, 1974; Führer & Chen, 1979; Gehrken, 1985; Schopf, 1985; Sieber & Benz, 1985; Langor & Raske, 1987a, b; Schopf, 1989; Hansen *et al*., 2001a; Doležal & Sehnal, 2007; Lester & Irwin, 2012; McKee & Aukema, 2015; Gent *et al*., 2017). In addition, voltinism of both species is influenced by differing diapause strategies.

In this review, we synthesize, summarize and compare available information on dormancy, overwintering biology and voltinism of these two important insects of spruce‐dominated forest ecosystems. We also discuss the influence of these life history strategies on population dynamics and the potential influence of a changing climate on future spruce bark beetle‐caused tree mortality worldwide.

## Ips typographus life history

*Ips typographus* males initially colonize a host tree and attract conspecifics using aggregation pheromones. It is a polygamous species, in which one male usually mates with two to three females. One female beetle deposits up to 80 eggs in excavated egg niches next to the mother gallery. After larval and pupal development, teneral beetles (i.e. freshly eclosed brood adults in the process of cuticle sclerotization prior to emergence from their natal host tree) feed on phloem tissue until maturation and subsequently emerge to colonize new hosts. In warm years with hot summers, some *I. typographus* populations, especially in Central Europe at low elevations, have the potential to establish up to three generations per year, resulting in swarming in spring and summer (Postner, 1974; Wermelinger, 2004; Wermelinger *et al*., 2012). However, only under extreme warm autumn conditions, a third generation may develop successfully into the overwintering adult stage before the cold period starts (Baier *et al*., 2007; Jönsson *et al*., 2011). Moreover, the establishment of sister broods (i.e. an additional generation produced by re‐emerging parental beetles) can contribute to population growth (Annila, 1969; Bakke, 1983; Anderbrant & Löfqvist, 1988; Anderbrant, 1989; Wermelinger & Seifert, 1999). *Ips typographus* usually attacks weakened Norway spruce affected by storms, snow, ice or drought. From the middle of the 20th to the beginning of the 21st Century, European forests were heavily affected by this beetle (Merker, 1957; Thalenhorst, 1958; Austarå *et al*., 1983; Bakke, 1983; Schelhaas *et al*., 2003; Wermelinger, 2004). For example, 30 million m^3^ of spruce were killed in southern Germany between 1944 and 1951 (Wellenstein, 1954), 5 million m^3^ from 1971 to 1981 in southern Norway (Bakke, 1989), 4 million m^3^ between 2006 and 2011 in Sweden (Kärvemo, 2015) and more than 16 million m^3^ from 2003 to 2010 in Austria (Krehan *et al*., 2010). Strong storms and drought provided vast amounts of suitable breeding material leading to a quick rise of beetle population densities (Schroeder & Lindelöw, 2002; Faccoli, 2009; Schroeder, 2010; Marini *et al*., 2012, 2013, 2017). Climate change will likely lead to a higher frequency of extreme weather events (IPCC, 2013), increasing the chance of bark beetle‐caused disturbances (Kausrud *et al*., 2012).

## Dendroctonus rufipennis life history

Although adult *D. rufipennis* may be observed flying from May through October, most tree attacks occur in early summer (Holsten *et al*., 1999), soon after temperatures become favourable for flight (Dyer, 1973; Safranyik & Linton, 1987; Holsten & Hard, 2001). It is a monogamous species with females initiating attacks on host trees and, after mating, they can deposit more than 100 eggs in two to five groups alternating on either side of the vertical mother gallery. A semivoltine life cycle (i.e. 2 years to complete a single generation) is typical throughout most of the species' range given average weather (Schmid & Frye, 1977). Under this cycle, development proceeds through the egg and four larval instars, with the first winter passed as a diapausing prepupa (Hansen *et al*., 2001a). Pupation occurs approximately 1 year after host infestation followed by eclosion to teneral adults (Massey & Wygant, 1954). The second winter is passed as a teneral adult either in pupal chambers or at the base of host trees (Massey & Wygant, 1954; Knight, 1961). After the second winter, sexually mature adults emerge and disperse to attack new host trees, completing the 2‐year cycle.

In years and locations with warm summers, a univoltine life cycle (i.e. one generation per year) has been observed in Colorado (Massey & Wygant, 1954; Knight, 1961), Utah (Hansen *et al*., 2001b), the Pacific Northwest coast (Schmid & Beckwith, 1975), British Columbia (Dyer, 1969) and Alaska (Werner & Holsten, 1985). Under this cycle, favourable temperatures accelerate development such that the prepupal diapause is averted and pupation and eclosion to teneral adults occur before onset of the first winter. These new brood adults then emerge and attack new hosts early the next summer, although their emergence may be delayed by up to 2 weeks compared with semivoltine beetles (E. M. Hansen, unpublished observations). A 3‐year life cycle has been observed at cold sites such as at high‐elevation, northerly aspect stands that are well‐shaded (Knight, 1961; McCambridge & Knight, 1972). Under this cycle, attacks on host trees may occur relatively late and/or larval development is slowed by cool conditions resulting in the first winter passed as an early‐instar larva. As cool conditions prevail during the second summer, there is inadequate heat for pupation and the second winter is passed as a prepupa. Pupation and eclosion to teneral adults occur during the third summer and new brood adults emerge the next summer. A 4‐year cycle has also been reported but observations were confounded by continued oviposition on hosts over consecutive years (McCambridge & Knight, 1972).

*Dendroctonus rufipennis* prefers felled hosts regardless of population phase, although eruptive populations predominantly colonize live, healthy trees (Wallin & Raffa, 2004). Large outbreaks often occur after disturbance events such as blowdown or logging operations, which allow rapid population growth in easily colonized material (Schmid & Frye, 1977), although threshold values of additional factors must also be surpassed. These include stand hazard, drought and favourable temperatures (Reynolds & Holsten, 1994; DeRose & Long, 2012; DeRose *et al*., 2013). Major outbreaks have occurred throughout the North American host tree range but particularly in the western part of the continent. An outbreak in Alaska during the 1990s impacted approximately 1.2 million hectares of spruce (Werner *et al*., 2006) and an ongoing outbreak in Colorado has affected more than 0.7 million hectares (U.S. Forest Service, 2015).

## Seasonality, dormancy and overwintering

Phenology of both *I. typographus* and *D. rufipennis* is strongly shaped by seasonal events and fluctuations of resources. Therefore, it is crucial for an individual to time its seasonal occurrence to exploit resources efficiently and to increase the chance of survival during harsh conditions. One strategy to ensure appropriate timing with seasonal events is to undergo a developmental delay by entering a dormant state. The developmental delay achieves synchrony and sets voltinism, and the dormant stage may additionally enhance survival during adversity by an increased stress resistance through physiological and behavioural preparations and by saving energy reserves through metabolic suppression (Lees, 1956; Tauber & Tauber, 1976; Tauber *et al*., 1986; Danks, 1987, 2006; Koštál, 2006; Hahn & Denlinger, 2007, 2011; Bentz & Jönsson, 2015).

Dormancy is a general term for a developmental and metabolic suppression and is further divided into diapause and quiescence. Diapause is a genetically programmed, hormonally controlled response to adverse conditions and not an immediate, direct reaction to a limiting factor (i.e. quiescence) (Tauber *et al*., 1986; Danks, 1987; Denlinger, 2002; Koštál, 2006). Diapausing insects are not developmentally static; they develop through three main phases: (i) induction; (ii) maintenance; and (iii) termination (Koštál, 2006). Furthermore, diapause can be induced regardless of environmental signals (i.e. obligate), or flexibly induced dependent upon environmental cues (i.e. facultative) (Koštál, 2006). Facultative and obligate diapause strategies may vary both among and within species. Within species, geographical variation in diapause response allows for the efficient exploitation of resources for development and reproduction in different environments, especially in species with wide distributions (Hodek & Okuda, 1993; Winterhalter & Mousseau, 2007; McKee & Aukema, 2015; Posledovich *et al*., 2015). Moreover, different dormancy strategies can also help to spread risks when uncommon and unpredictable environmental changes occur (Tauber *et al*., 1986; Danks, 1987).

In addition to dormancy, many bark beetle life history events are temperature‐driven, including developmental rates and thresholds, survival, thresholds for the onset of swarming or feeding, and thermal sums to complete ontogenetic phases (Bakke, 1968; Bentz *et al*., 1991; Lobinger, 1994; Coeln *et al*., 1996; Wermelinger & Seifert, 1998, 1999; Doležal & Sehnal, 2007; Gent *et al*., 2017; Schebeck & Schopf, 2017). Because winter temperatures in temperate regions frequently drop below the melting point of water, temperature also plays a major role in overwintering survival, and cold adaptations are often related to the expression of dormancy (Zachariassen, 1982; Hodek & Hodková, 1988; Denlinger, 1991; Bale, 1996; Sømme, 1999; Sinclair *et al*., 2003a, 2003b; Danks, 2006; Lee, 2010).

## Diapause and overwintering of I. typographus

Various *I. typographus* populations at different latitudes and altitudes can establish a different number of generations each year (Seitner, 1923; Kuhn, 1949; Wild, 1953; Wellenstein, 1954; Thalenhorst, 1958; Annila, 1969; Forsse, 1991). Central European lowland populations were found to be mostly bivoltine (i.e. two generations per year) and montane, subalpine and northern European populations were observed to be mainly univoltine. However, field observations reported an absence of *I. typographus* reproductive behaviour during times of permissive environmental conditions and only the presence of immature adults late in the year (Seitner, 1923; Wild, 1953; Merker, 1957; Merker & Adlung, 1958; Annila, 1969). For example, some populations in northern latitudes were found to be strictly univoltine, even under favourable temperature conditions for development (Annila, 1969).

Controlled laboratory studies on the influence of photoperiod and temperature on the development of gonads and flight muscles, on physiological parameters (including respiration rates and cold hardiness) and on emergence and dispersal support an adult facultative reproductive diapause (Schopf, 1985, 1989; Doležal & Sehnal, 2007). Before *I. typographus* enters diapause, it is able to establish new generations, thus having the potential for multivoltinism (i.e. multiple generations per year). Several laboratory and field studies suggest that a second phenotype, an obligate diapause, might also be expressed (Schopf, 1985, 1989; Netherer, 2003; Doležal & Sehnal, 2007; Dworschak, 2013; Schroeder & Dalin, 2017; N. Dobart & A. Schopf, unpublished observations). Clear evidence to distinguish between a facultative and obligate diapause could be informed through further physiological and behavioural observations (e.g. experiments at additional photoperiod and temperature conditions), but also through genomic and transcriptomic studies.

Adult facultative diapause is induced by photoperiod, modified by temperature (Schopf, 1985, 1989; Doležal & Sehnal, 2007). The critical day length varies depending on the geographical origin of a population, wherein life cycles are matched with environmental conditions. For example, beetles from Central European lowlands express a facultative diapause that is induced by a day length shorter than 15 h (Schopf, 1989; Doležal & Sehnal, 2007) and temperatures below constant 23 °C (Doležal & Sehnal, 2007). Sensitive stages for diapause induction occur late in the beetle's life cycle (i.e. third‐instar larva, pupa and adult); the development of earlier ontogenetic stages appears to be independent of photoperiod (Doležal & Sehnal, 2007). In more northern regions, diapause is induced by a longer critical day length. Along a latitudinal gradient in Sweden, beetles from a more northern origin have a critical day length of approximately 19 h, and those from a more southern population have a critical day length of approximately 16–17 h. Furthermore, diapause incidence is generally higher in more northern latitudes (Schroeder & Dalin, 2017). Termination of facultative diapause under natural conditions requires a certain chilling period that occurs in December/January in Central Europe. After diapause termination, *I. typographus* enters a quiescent state until conditions are permissive for gonad development and reproduction (Doležal & Sehnal, 2007).

In environments with thermal conditions that lead to a univoltine rather than a multivoltine life cycle, an obligate diapause may have evolved (Schroeder & Dalin, 2017). A proportion of beetles from some Central European populations neither emerged from breeding systems, nor dispersed, and gonads did not mature when they were exposed to permissive photoperiods and temperature conditions in the field and in the laboratory (Schopf, 1989; Netherer, 2003; Dworschak, 2013; N. Dobart & A. Schopf, unpublished observations). Adults remaining under the bark of logs at long‐day conditions in the laboratory had lower metabolic rates than emerged beetles (Schopf, 1989). Thus, behavioural and physiological traits suggest that beetles expressed diapause although favourable environmental conditions prevailed. Some *I. typographus* from specific Scandinavian populations also enter diapause although exposed to photoperiods above the critical day length (Doležal & Sehnal, 2007; Schroeder & Dalin, 2017), including conditions >23 h light, with higher diapause ratios at a given day length in more northern regions (Schroeder & Dalin, 2017). However, the underlying mechanisms, timing of entering and terminating diapause, and influences of other environmental factors (e.g. nutritional value of the phloem tissue) are still unknown. Laboratory studies suggest that this diapause pathway could be terminated by a certain period of cool temperatures, comparable to the termination of facultative diapause (Doležal & Sehnal, 2007).

Although populations might harbour both facultative and obligate diapause phenotypes, the occurrence of each type could depend on the geographical origin of a population. An obligate diapause phenotype would predominantly be found in higher latitudes and altitudes, whereas a facultative phenotype would mainly be found in more southern regions and lower elevations (N. Dobart & A. Schopf, unpublished observations).

Furthermore, responses to diapause‐inducing signals might face selective pressures and evolutionary changes might occur quickly. Diapause expression at a given photoperiod was found to differ between field caught beetles and individuals reared in the laboratory for many generations (Doležal & Sehnal, 2007; Schroeder & Dalin, 2017).

In addition to entering diapause, overwintering success at low temperatures is affected by physiological and behavioural adaptations to cope with freezing of body fluids. *Ips typographus* exhibits enhanced cold resistance when it develops at short day length in the laboratory (Schopf, 1985). Furthermore, metabolic suppression during diapause serves to save energy reserves during hibernation and increases the chance of survival (Schopf, 1989; Dworschak *et al*., 2014). However, low temperatures directly control successful overwintering of *I. typographus*. This species is freeze avoidant and dies when ice forms in body fluids. By accumulating cryoprotectant substances, a mixture of sugars and polyols that lower the melting point of the haemolymph, body liquids can remain fluid although the ambient temperature falls below the melting point of water (Koštál *et al*., 2007, 2011). This mechanism is defined as supercooling, and the temperature at which ice formation occurs is the supercooling point (SCP) (Zachariassen & Kristiansen, 2000; Lee, 2010). SCPs follow a seasonal trend where the lowest values for adults are recorded in mid‐winter, approximately −25 °C in Central Europe (Schopf & Kritsch, 2010; Koštál *et al*., 2011) and as low as −32 °C at more northern latitudes (Annila, 1969). In general, pre‐imaginal stages have higher SCPs than adults, and pupae are more resistant to cold than larvae (Annila, 1969; Schopf & Kritsch, 2010). The effects of cryoprotectants are reflected by a seasonal trend as well, similar to that of SCPs (Koštál *et al*., 2011). Feeding behaviour also influences resistance to cold. To increase the chance of overwinter survival, adults empty the gut before hibernation to get rid of potential ice nucleators (Annila, 1969) that can cause substantial overwinter mortality in larvae (Schopf & Kritsch, 2010).

In northern Europe, where temperatures are generally colder than in more southern latitudes, adult beetles leave the brood tree in summer and autumn to overwinter in habitats insulated by snow including in litter, soil, moss or under the bark of fallen trees (Annila, 1969). In Central Europe, *I. typographus* mainly overwinters under the bark of host trees (Harding & Ravn, 1985; Faccoli, 2002; Schopf & Kritsch, 2010; Wermelinger *et al*., 2012), although the proportion of beetles hibernating outside of trees increases with increasing elevation (Wermelinger *et al*., 2012). In Central European lowlands, only a small ratio of adult beetles hibernates outside the brood tree in the forest litter when ambient conditions, such as phloem quality, are unfavourable for successful overwintering (Biermann, 1977).

Regardless of the overwintering site, the ontogenetic stage for successful hibernation is the adult (Annila, 1969; Harding & Ravn, 1985; Faccoli, 2002; Schopf & Kritsch, 2010), although pre‐imaginal stages can survive high sub‐zero temperatures (Štefková *et al*., 2017). Hibernation below the snowpack, where temperatures are approximately 0 °C, can increase survival, particularly in the adult stage (Annila, 1969). In the Southern Alps, although winter temperatures did not fall below −12 °C, pre‐imaginal stages did not survive and, regardless of the ontogenetic stage, approximately half of individuals did not hibernate successfully (Faccoli, 2002). Similar results were found in an Austrian lowland population in which all larvae and pupae died, and adult mortality was approximately 50%, although temperatures were higher than −10 °C (Schopf & Kritsch, 2010).

In Southern Germany, almost all young adults survived winter and emerged in spring when they had fed before cold conditions to slightly enhance their lipid content to synthesize cryoprotectants or to maintain metabolism. Highest mortality rates were found among larvae, although winter was relatively mild with lowest temperatures of −12 °C (Dworschak *et al*., 2014). To avoid mortality as a result of inoculative freezing (Koštál *et al*., 2011), adults seek relatively dry microhabitats for hibernation, such as dry litter or outer parts of the bark (Bender, 1948; Wild, 1953; Schopf & Kritsch, 2010), whereas immobile pre‐imaginal stages are more likely to die from external ice formation (Schopf & Kritsch, 2010).

## Diapause and overwintering of D. rufipennis

Temperature has repeatedly been correlated with *D. rufipennis* developmental rates, diapause induction and subsequent voltinism (Dyer *et al*., 1968; Dyer, 1970; Dyer & Hall, 1977; Werner & Holsten, 1985; Hansen *et al*., 2001a; Berg *et al*., 2006; Hansen *et al*., 2011). Dyer *et al*. (1968) estimated a lower developmental threshold of about 6 °C for all life stages whereas data from Hansen *et al*. (2001a) suggest subtle differences in lower thresholds among life stages. Various proportions of tested individuals completed most life stages at 5.5 °C, whereas the threshold for egg hatch was estimated as 7–9 °C. Therefore, temperature‐related quiescence should prevent development during winter and we speculate that this is the physiological mechanism for early‐instar overwintering larvae among *D. rufipennis* developing on the 3‐year cycle.

In contrast to quiescent larvae, prepupae and teneral adults overwinter in diapause. Several lines of evidence indicate that *D. rufipennis* prepupal overwintering results from a facultative diapause induced by relatively cool temperatures experienced during the third and fourth instars (Dyer, 1970; Dyer & Hall, 1977; Hansen *et al*., 2001a, 2011). For example, there is a discontinuous relationship between developmental rate and temperature in the fourth instar, suggesting that morphological and diapause development occur simultaneously during this life stage (Hansen *et al*., 2001a, 2011). We consider this diapause facultative because developmental delays (i.e. extended voltinism) are averted if temperatures during the first summer are sufficient for pupation.

Photoperiod has no measurable effect on prepupal diapause induction in *D. rufipennis*, which spends almost its entire life cycle in a subcortical environment (Hansen *et al*., 2011). In a study using *D. rufipennis* from Utah (38°37.424′N; 111°59.188′W; 3130 m elevation), larvae were reared at constant 18 °C (diapause‐averting) or 12 °C (diapause‐inducing) under short‐day (LD 8 : 16 h) or long‐day conditions (LD 16 : 8 h). At 18 °C, total time to pupation did not significantly differ between the photoperiodic treatments (approximately 27 days) whereas none of the larvae reared at 12 °C pupated regardless of photoperiod after 100 days when the experiment was ended (lower threshold for pupation ≤5.5 °C). Instead, diapause has been induced in the laboratory by temperatures below 13.5–15 °C during the third through late fourth instars and cool temperature exposure during the early fourth instar appears to be required for diapause‐related developmental delays (Dyer & Hall, 1977; Hansen *et al*., 2001a, 2011). Prepupal diapause induction in *D. rufipennis* is not simply a switch that causes diapause to be expressed or not. Instead, diapause is a continuous process (Danks, 1987) and diapause‐related developmental delays in *D. rufipennis* can range from just a few days to several weeks, an interval that appears sufficient to prevent pupation just before the onset of winter. The degree of delay is related to the duration of temperatures below the induction threshold, whereas the absolute temperature itself has little, if any, detectable effect (Hansen *et al*., 2011).

Teneral adults overwinter in reproductive diapause. Massey & Wygant (1954), studying *D. rufipennis* from Colorado, found evidence that adult overwintering may be required to attain sexual maturity. Although they did not show supporting evidence, Safranyik *et al*. (1990) also observed that *D. rufipennis* (presumably from Canada) overwintering as an adult is mandatory for reproduction regardless of life cycle duration and called this ‘diapause’. Attempts to rear successive generations of *D. rufipennis* from Utah at room temperature (approximately 21 °C) were abandoned when the beetles did not emerge from infested bolts more than 100 days after eclosion to the adult stage (E. M. Hansen & B. J. Bentz, unpublished observations). In comparison, the entire life cycle (oviposition through adult emergence) of *Dendroctonus ponderosae* Hopkins, which has broadly similar life stage‐specific developmental rates compared with *D. rufipennis* (Bentz *et al*., 1991; Hansen *et al*., 2001a), can be completed in approximately 70 days under those same conditions (Bracewell *et al*., 2013). However, diapause developmental delays in *D. rufipennis* may vary with geography. A proportion of adults from New Mexico (36°43.287′N; 106°15.391′W; 3034 m elevation) and Utah (40°45.660′N; 110°52.980′W; 2885 m elevation) successfully reproduce without overwintering, particularly beetles from the more southerly population (E. M. Hansen & B. J. Bentz, unpublished observations). Geographical variation in the ratio of facultative and obligate adult diapause is therefore likely and also suggests the potential for geographical variability in prepupal diapause capacity.

*Dendroctonus rufipennis* larvae and prepupae overwinter in the bole near the parent egg gallery. New adults overwinter in their pupal chambers under the bark or emerge to overwinter in the duff or near the root collar of their natal host. Parent beetles frequently survive to produce sister broods in the same or the next year as determined from collections of adult beetles from logs or trees in which all broods consisted of immature stages (Massey & Wygant, 1954; Hansen & Bentz, 2003). These adults overwinter in their egg galleries, at the base of their host tree or in new galleries on a fresh host. Beetles overwintering below the snowline are afforded considerable protection from possible lethal winter temperatures and from woodpecker predation. In Colorado, Knight (1958) observed *D. rufipennis* mortality as high as 98% from woodpecker foraging. Similarly, extreme cold causes substantial *D. rufipennis* mortality, even to the point of halting epidemic population outbreaks. Temperatures as low as −49 °C were associated with the loss of an estimated 75% of overwintering *D. rufipennis* in Colorado (Massey & Wygant, 1954), whereas a record low temperature of −40 °C was associated with the loss of 88% of the local population in Arizona overwintering above the snow level, as estimated from north and south subcortical samples from 98 trees over 20 locations (Frye *et al*., 1974). Minimum temperatures are typically below −40 °C in central Alaska, making survival above the snowline unlikely (Miller & Werner, 1987). Therefore, the semivoltine cycle, with overwintering prepupae unable to move to protected microhabitats, will result in low survival probability whereas teneral adults on the univoltine cycle can emerge from their pupal chambers and overwinter below the snowpack. Therefore, univoltine *D. rufipennis* have considerably reduced exposure to these two important mortality agents.

As with *I. typographus*, *D. rufipennis* is reliant on supercooling to survive temperatures below the melting point of water. Using field‐conditioned adults and larvae collected from Colorado in January or March/April, Massey & Wygant (1954) found that most adults suffered mortality at −26 °C, regardless of collection timing, whereas most larvae did not die in the test until temperatures were lowered to −32 °C or colder. In contrast, Miller & Werner (1987) found that adults and larvae collected from central Alaska had similar SCPs that varied seasonally, ranging from −12 °C during summer to −31 °C during winter. The species is also freeze avoidant and relies on glycerol synthesis to achieve SCPs that ensure some portion of the population survives unusually cold winters, with individuals overwintering below the snowline having enhanced odds of survival (Miller & Werner, 1987). In comparison, the pupal stage of *D. ponderosae* is considered to be one of the most susceptible stages to cold‐induced mortality (Amman, 1973) and field sampling has found *D. rufipennis* pupae as late as mid‐October but rarely during winter (E. M. Hansen & B. J. Bentz, unpublished observations). We therefore hypothesize that prepupal diapause is a mechanism to ensure that overwintering occurs in a less susceptible life stage.

## Implications of diapause and overwintering on phenology, voltinism and outbreaks

Increased temperature and a higher chance of extreme weather events associated with climate change (IPCC, 2013) could enhance the probability of abiotic and biotic disturbances in some forests (Dale *et al*., 2001; Berg *et al*., 2006; Bentz *et al*., 2010; Bentz & Jönsson, 2015). Observations over the last decades, as well as models, found alterations of phenology and distribution patterns of several bark beetle species (Lange *et al*., 2006; Jönsson *et al*., 2007, 2009, 2011; Faccoli, 2009; Bentz *et al*., 2010, 2016). In addition to direct effects (i.e. the influence of temperature on development and survival) of a changing climate, indirect factors may also affect bark beetles, including alterations of host tree susceptibility and availability, competition with other species sharing the same resources, and altered interactions with organisms of various trophic levels (Bentz *et al*., 2010; Addison *et al*., 2015; Økland *et al*., 2015; Schroeder & Dalin, 2017).

For *I. typographus*, recent studies found an earlier onset of swarming and thus the potential to establish more generations per year, with predictions of a shift from uni‐ to bivoltinism or from bi‐ to trivoltinism in parts of Europe (Faccoli, 2009; Jönsson *et al*., 2011). With higher temperatures, the lower developmental threshold could be exceeded earlier and for longer periods in a year, therefore more often surpassing the thermal sums required for the development of a full generation (Wermelinger & Seifert, 1998; Baier *et al*., 2007). In Europe, however, the development of more than three entire generations per year is considered unlikely (Hlásny & Turćáni, 2009). Similarly, predictions for *D. rufipennis* suggest that warming temperatures will result in a shift from semi‐ to univoltinism over most of its range as the prepupal diapause is averted (Bentz *et al*., 2010), although bivoltinism could be restricted by an obligate adult reproductive diapause. Under controlled temperatures in the laboratory, *I. typographus* development from egg to pupation was faster than *D. rufipennis* (Wermelinger & Seifert, 1998; Hansen *et al*., 2001a), confirming the increased likelihood for multivoltinism in *I. typographus* with warming temperatures. For both species, however, as temperatures exceed optimal development thresholds (i.e. approximately 25 °C for *D. rufipennis* and 30 °C for *I. typographus*), the developmental rate will slow (Wermelinger & Seifert, 1998; Hansen *et al*., 2011). Without evolutionary changes in developmental rates, continued warming beyond upper development thresholds will eventually result in conditions that constrain development and cause death.

Temperature also influences voltinism of both species through its effect on diapause. When the adult *I. typographus* diapause is facultative, new generations can be initiated until inducing cues in late summer are perceived (Schopf, 1989; Doležal & Sehnal, 2007), thus contributing to the increased likelihood of multivoltinism as temperatures warm (Jönsson *et al*., 2011). Similarly, a facultative prepupal diapause in *D. rufipennis* that is averted with warm summer temperatures will increase the likelihood of univoltinism (Hansen *et al*., 2001a, 2011). An adult obligate diapause in both species, however, could limit generation times to a strictly univoltine life cycle, despite thermal conditions favourable for development of additional generations. Assuming a mix of facultative and obligate diapausers in a population, however, the ratio of each could change as warming temperatures invoke selective pressures that favour multivoltinism and hence a facultative diapause (Danks, 1987). In addition, facultative diapausers from more southern regions could migrate northwards and lead to shifts in the composition of phenotypes (Jönsson *et al*., 2011).

The production of sister broods can also influence the population dynamics of both species (Schmid & Frye, 1977; Anderbrant & Löfqvist, 1988; Anderbrant, 1989). In *I. typographus*, the presence of sister broods in a changing climate could be more likely because parent adult re‐emergence, brood establishment and development are dependent on warm conditions that may be more often met in warmer climates (Annila, 1969; Anderbrant, 1986, 1989). Sister broods are also common in *D. rufipennis* (Massey & Wygant, 1954) and may lead to rapid population increase (Hansen & Bentz, 2003), although more studies will be necessary to confirm this prediction. Population growth of univoltine *I. typographus* populations, however, will likely benefit more from sister broods than growth of multivoltine populations (Wermelinger & Seifert, 1999). In both species, assessment of sister broods and the number of generations in the field using, for example, pheromone trapping data can be difficult, as a result of a lack of distinguishing characteristics among cohorts from the same generation, as well as sister broods and generations from a previous year.

*Dendroctonus rufipennis* can overwinter successfully as diapausing prepupa or diapausing adult, or as a quiescent larva in the case of the 3‐year life cycle (McCambridge & Knight, 1972; Hansen *et al*., 2001a). In contrast, only *I. typographus* adults can survive low sub‐zero temperatures successfully (Annila, 1969; Harding & Ravn, 1985; Faccoli, 2002; Baier *et al*., 2007; Schopf & Kritsch, 2010; Dworschak *et al*., 2014). Because diapause is at least modulated by temperature, warm late summer temperatures could influence both species although in different ways. In *I. typographus*, warm temperatures that postpone the adult diapause induction (Doležal & Sehnal, 2007; Jönsson *et al*., 2011) could allow a prolonged season for development and reproduction, with limited time for brood development and preparation for hibernation. Warm late summer temperatures would allow *D. rufipennis* to avert a prepupal diapause and therefore spend the first winter as an adult, resulting in a univoltine rather than semivoltine life cycle. Detrimental effects could also occur, however, if development proceeds to a life stage that is not resistant to severe cold, such as the pupal stage in *D. rufipennis* and the pre‐imaginal stages in *I. typographus*. Prepupal (*D. rufipennis*) and adult (*I. typographus*) diapause are therefore ‘safety’ strategies to prepare for an early and abrupt onset of unfavourable conditions and to reduce the risk of mortality.

## Conclusions and outlook on future research

The two bark beetle species reviewed here, *I. typographus* and *D. rufipennis*, are major disturbance agents in spruce forests globally and they employ similar strategies to survive extreme winter conditions, including diapause and resistance to cold. Differences in life stage‐specific developmental rates and diapausing life stages, however, result in different phenologies with potential varying effects on voltinism and population outbreak dynamics (Table 1).

**Table 1:** Species specific and shared characteristics of Ips typographus and Dendroctonus rufipennis with focus on diapause and overwintering biology.

Characteristic	*Ips typographus*		*Dendroctonus rufipennis*
	Species specific	Shared	Species specific
Natural distribution	Eurasia	–	North America
Host trees	Main host *Picea abies*	–	All *Picea* spp. in its natural range
Voltinism	Uni‐ to multivoltine, up to three generations per year	–	Mainly semi‐ or univoltine, a 3‐year life cycle is possible at very cool sites
Mating and reproduction	Male initiates attack, polygamous (usually two or three females), up to 80 eggs/female	–	Female initiates attack, monogamous, more than 100 eggs/female
Pheromone communication	–	Aggregation pheromones (for both sexes) for mass attack	–
Outbreaks	–	After abiotic disturbances (e.g. storms, snow, ice, and drought)	–
Diapause	–	Adult reproductive diapause that is supposed to be either facultative or obligate	Facultative prepupal diapause
Diapause inducing cues	Photoperiod (modified by temperature) induces facultative diapause	Obligate diapause expressed regardless of environmental signals	Temperature induces prepupal, facultative diapause
Type of cold hardiness	–	Freeze avoidant	–
Overwintering stage	Usually adult	–	Prepupa, adult^a^
Overwintering site	–	Under the bark of host trees or on the ground of forests	–

a In uncommon cases of a 3‐year life cycle larvae may overwinter.

Both species can overwinter as a diapausing adult and, for *I. typographus*, this is the only life stage considered resistant to harsh winters. *Dendroctonus rufipennis*, in contrast, can also successfully overwinter as a diapausing prepupa or, less commonly, as a quiescent larva. Facultative and obligate adult diapause phenotypes that vary geographically are assumed for *I. typographus* and have recently also been found in *D. rufipennis*. Similar to the variation in adult diapause found in the closely related *Dendroctonus simplex* LeConte (McKee & Aukema, 2015), facultative diapause phenotypes are most prevalent in the southernmost part of each species' distribution. Although understanding the basis for variability among populations of these species requires additional investigation, phenotypic plasticity in diapause capacity is undoubtedly a local adaptation that mediates voltinism. For example, in *I. typographus* populations with predominately facultative rather than obligate adult diapause phenotypes, bivoltinism is possible with the potential for multivoltinism in a warming climate. Bivoltinism is currently common in southern latitude and low elevation *I. typographus* populations but has not been recorded in *D. rufipennis*. The likelihood of bivoltinism in *D. rufipennis* with climate warming is uncertain because of relatively colder habitats and slower life stage‐specific developmental rates. In the coldest locations where both species may have a predominance of obligate adult diapause phenotypes, *I. typographus* populations are currently restricted to a univoltine life cycle, whereas *D. rufipennis* has a semivoltine (or longer) life cycle. A warming climate, however, may increase selection for the facultative phenotype, potentially leading to bivoltinism in *D. rufipennis* and range expansion of that life cycle in *I. typographus*.

The number of generations produced per year may have a large impact on tree mortality caused by *I. typographus* and *D. rufipennis*. Understanding the factors that influence variability in voltinism across environments, including local adaptation in diapause strategies, is therefore needed for making robust predictions across the vast ranges of both species. The influence of additional diapause terminating cues (besides temperature), including photoperiod, is still not sufficiently clear for *I. typographus* but is critical for predicting the onset of spring swarming and subsequent generation timing as winters warm. Although *D. rufipennis* adult and prepupal diapause development appears to be terminated by cold temperatures experienced in early winter, altered thermal patterns will shift the timing of adult emergence, potentially modifying the life cycle duration of the subsequent generation. Understanding similar and differing climate effects on the life history strategies of each species will inform predictions of landscape‐scale impacts and global range expansion in a changing climate.
